# Bladder cancer stem cells: clonal origin and therapeutic perspectives

**DOI:** 10.18632/oncotarget.19112

**Published:** 2017-07-08

**Authors:** Yi Li, Kaisu Lin, Zhao Yang, Ning Han, Xiaofang Quan, Xiangyang Guo, Chong Li

**Affiliations:** ^1^ Department of Anesthesiology, Peking University Third Hospital, Beijing, China; ^2^ Department of Oncology, the Affiliated Aoyang Hospital of Jiangsu University, Zhangjiagang, China; ^3^ Core Facility for Protein Research, Institute of Biophysics, Chinese Academy of Sciences, Beijing, China; ^4^ Department of Life Science and Technology, China Pharmaceutical University, Nanjing, China; ^5^ Beijing Jianlan Institute of Medicine, Beijing, China

**Keywords:** bladder cancer, cancer stem cell, BCSCs

## Abstract

In this article, we review the origin and therapeutic perspectives of bladder cancer stem cells (BCSCs), which are integral to the initiation, high recurrence and chemoresistance of bladder cancer. BCSCs are heterogenous and originate from multiple cell types, including urothelial stem cells and differentiated cell types, including basal, intermediate stratum and umbrella cells. Cell surface markers, including CD44, CD67LR, EMA, ALDH1A1 and BCMab1, are used to identify and isolate BCSCs. The Hedgehog, Notch, Wnt and JAK-STAT signaling pathways play key roles in maintaining the stemness, self-renewal and proliferative potential of BCSCs. High expression of ABC transporters, acetaldehyde dehydrogenase, antioxidants and apoptosis resistance proteins in BCSCs play a critical role in chemoresistance. Consequently, a greater understanding of the biology of BCSCs will be important for identifying effective therapeutic targets to improve clinical outcomes for bladder cancer patients.

## INTRODUCTION

Bladder cancer is the most common malignant tumor of urinary system with an increasing incidence [1]. Papillary and non-papillary carcinomas are the two different types of bladder cancer with unique, yet overlapping clinical and pathological features [2]. The urothelial carcinomas of the bladder are generally called bladder cancer or bladder transitional cell carcinoma and are most common. Nearly 80% of urothelial carcinomas are non-invasive urothelial papillomas with high recurrence rates after resection without infiltrating the bladder wall or distant metastasis. The rest 20% are muscle-invasive bladder cancers that are highly invasive with distant metastasis [3]. Although patients with superficial papillary lesions usually experience multiple recurrences, only 10%-30% of them develop into high-grade invasive tumors. On the contrary, most patients with high-grade invasive bladder cancers do not show superficial papillary lesions. The 5-year survival rate of muscle-invasive bladder cancer patients is low and has shown no improvement inspite of great advances in prognosis and surgical methods. The bladder cancer stem cells (BCSCs) are probably responsible for the high recurrence rates of bladder cancer, tumor heterogeneity and other complex biological behaviors.

The cancer stem cells (CSCs) are defined as a subset of cells with the ability to self-renew and differentiate into hierarchical tumor cells, thereby contributing to tumor heterogeneity [4]. Cancer stem cells (CSCs) were first identified in hematopoietic malignancies as a subgroup of cells that demonstrated stemness and differentiated into heterogenous populations of tumor cells [5, 6]. CSCs have also been identified in solid cancers like colorectal carcinomas [7]and ovarian [8] and liver cancers [9]. They also demonstrate self-renewal and differentiation properties [10] and are responsible for tumor heterogeneity and high recurrence rates of many cancers [4].

Bladder cancer is highly recurrent, metastatic and heterogenous, thereby resulting in poor prognosis [11]. It is postulated that bladder cancer stem cells are partly responsible for the clinical characteristics and complex biological behavior of bladder cancer [12]. Therefore, understanding the role of CSCs in bladder cancer is critical to gain insights into the mechanisms responsible for high recurrence and metastasis. Additionally, it would help identify novel therapeutic avenues and facilitate better prognosis. In this review, we focus on the new frontiers and progress in the study of bladder cancer stem cells.

## ORIGIN OF BLADDER CANCER STEM CELLS

### Bladder cancer types

The 2 types of bladder cancers, namely non-muscle and muscular bladder cancers originate from two different pathways [13]. Studies with transgenic mice revealed that normal stem cells with HRAS or FGFR3 mutations can transform into BCSCs that develop into superficial non-muscle invasive bladder cancer, whereas stem cells with p53/Rb/PTEN gene mutations transform into BCSCs that initiate muscular invasive bladder cancer [14]. Further, transgenic mice with continuous activation of HRAS gene developed non-invasive bladder cancer, whereas transgenic mice with UPII promoter-SV40T gene in combination with persistent inactivation of p53 and pRb gene developed invasive bladder cancer [15]. These studies postulated that BCSCs probably originated from transformed transgenic stem cells.

### The origin of bladder cancer stem cells

The topic about the origin of CSCs is still controversial [16, 17]. It is widely assumed that CSCs may arise from normal stem cells having suffered gene mutations [18] and the generation of CSCs from normal stem cells is complex [17]. By single cell sequencing, Yang *et al* showed that bladder cancer stem cells (BCSCs) originated from bladder cancer stem cells (BCSCs) or bladder cancer non-stem cells (BCNSCs) with clonal homogeneity among BCSCs and BESCs or BCSCs and BCNSCs [19]. Apart from stem cells, CSCs can also originate from progenitor cells or differentiated cells that undergo de-differentiation or tumor cells that gain stem cell properties or fusion cells [16].

**Figure 1:**
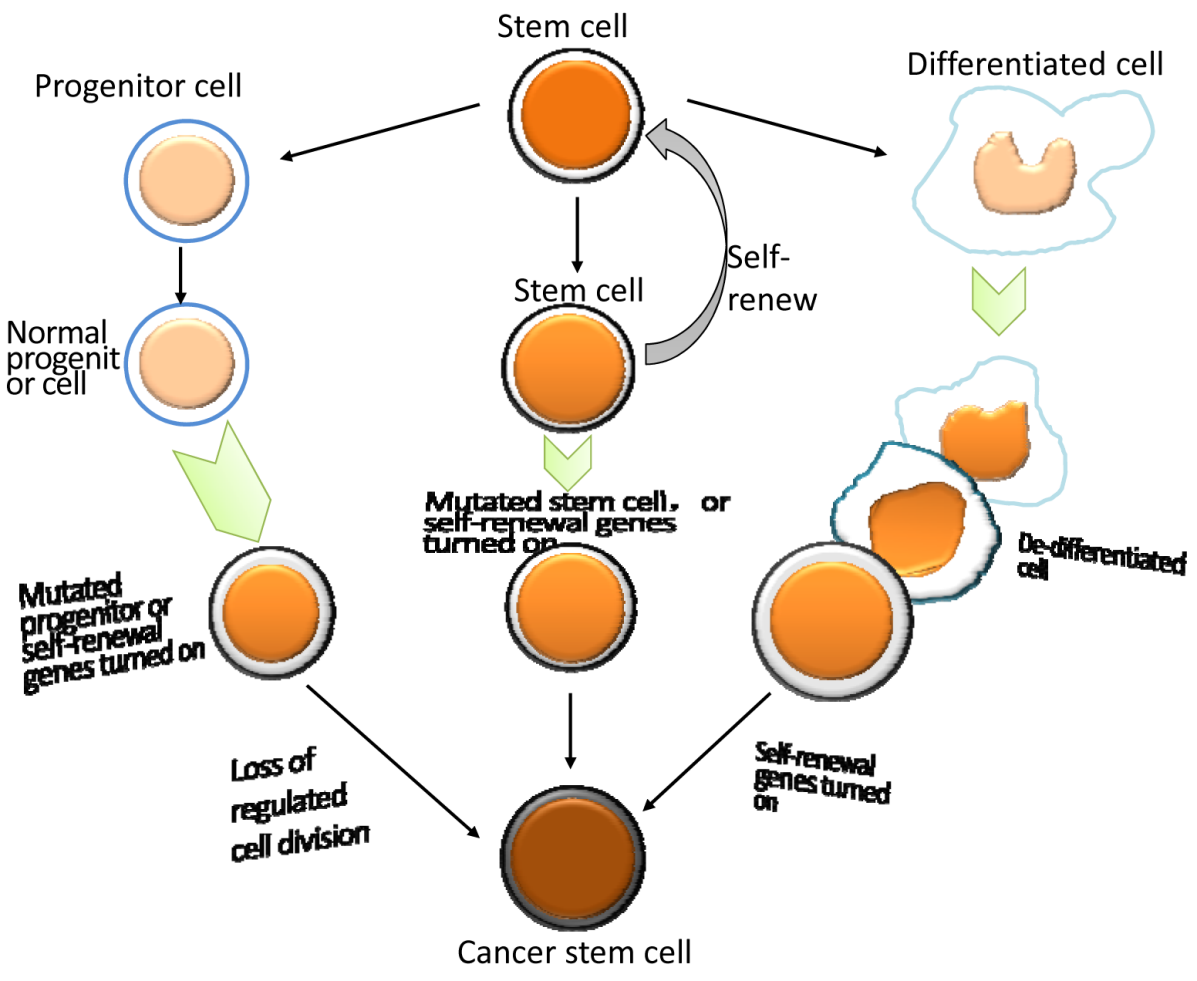
The origin of bladder cancer stem cells

Several common markers of BCSCs including CD44^+^, EMA^-^, 67LR^+^, BCMab1^+^ [20, 21]are located in the basal cell layer of bladder tumor leading to more debates regarding the source of BCSCs. Theoretically, if all the markers are from a specific cell type in bladder cancer, it is assumed that BCSCs may have originated from mutated normal stem cells. On the other hand, if the markers are expressed on different normal cell types, then the BCSCs may be derived from progenitors or differentiated cells that acquired de-differentiation properties due to mutations, thereby leading to different subgroups of BCSCs (Figure 1).

#### Normal urothelial stem cells

Colon cancer stem cells generally originate from intestinal epithelial stem cells expressing Lgr5 [22]. BrdU pulse-chase labeling assays suggested that the urothelial stem cells were located in the basal cell layer [23]. This was further confirmed by mitochondria DNA mutation experiments [24]. Nitrosamine induced bladder cancer model confirmed that invasive bladder cancer originated from stem cells from basal cell layer [25]. These studies suggested that BCSCs originated from urothelial stem cells in the basal cell layer.

#### Urothelial stem cell

Urothelial, bone marrow and adipose stem cells are all capable of repairing bladder damage [26]. Therefore, these stem cells are possible sources of BCSCs. The gram-negative bacterium, *Helicobacter pylori* is a carcinogen that recruits bone marrow derived cells (BMDCs) in gastric cancer [27]. However, in chemical induced bladder cancer, BMDCs are associated with inflammation in response to tumor and not related to tumorigenesis [28].

#### Basal cells

BCSCs were found to be CD44^+^CK5^+^CK20^-^ that were characteristic basal cell markers [5]. Yang *et al.* showed that CD44^+^ cells were in the basal cell layer of normal urothelium and urothelial carcinoma [20]. Shin *et al* demonstrated that muscular invasive bladder cancer were derived specifically from Sonic hedgehog (SHh) expressing basal cells [25].

#### Intermediate stratum cells

The cells within the intermediate layer express different levels of CD44, which has been identified also as the BCSC marker [20]. Lineage tracing experiments in a mouse tumor model demonstrated that papillary cancer cells mainly originated from the intermediate layer [20]. Further, Brant *et al* thought that mutations of the fibroblast growth receptor FGFR3 in intermediate layer cells might help intermediate stratum bladder cells transform into malignant low-grade papillary carcinoma and urinary epithelial hyperplasia [12]. These experiments showed that non-muscle invasive bladder stem cells may originate from the intermediate layer cells.

#### Umbrella cells

The muscular invasive bladder cancer of intracavity type showed aberrant expression of transcription factors PPARG, ESR1, and FGFR3 [22]. Also, they expressed umbrella cell markers such as keratin 20 [29]. This suggested that BCSCs that develop into muscle invasive bladder cancer are derived from umbrella cells.

#### Bladder cancer cells

Cancer stemness is affected by genotype heterogeneity, epigenetic alterations and tumor microenvironment [30]. The interaction of tumor cells with tumor associated fibroblasts, macrophages, perivascular stroma and endothelial cells is critical for their survival in hypoxic and low nutritional conditions. The CSC-like phenotype of bladder cancer is observed during late stages of tumor development suggesting that the early bladder cancer cells may transform into CSCs through mechanisms such as epithelial mesenchymal transformation (EMT), dedifferentiation, and hypoxia [31].

### Identification of bladder cancer stem cells

#### BCSC surface markers

Bladder cancer stem cells were identified for the first time in 2009 *via* the markers used to isolate normal stem cells [32]. So cell surface markers are traditionally used to isolate BCSCs. Since the biological behavior and phenotypes of tumor cell lines may have changed due to long-term *in vitro* culturing, primary or early passage tumor cell lines are ideal for isolating and identifying of BCSCs.

Chan *et al.* demonstrated that 40% of more than 300 bladder transitional cell carcinoma patient samples contained CD44^+^ cells. They further showed CD44 expressing subpopulation of cells in serial xenograft experiments with fresh patient samples and the tumorigenicity of CD44^+^ bladder cancer cells was found to be 10-200 times higher than CD44^-^ bladder cancer cells when transplanted in immunodeficient mice [5]. The CD44^+^ cells maintained heterogeneity of the primary tumor after sequential transplantation, thereby complying with the functional standard for stem cells [5]. *In vitro* clonal sphere formation studies have been widely used to evaluate the stemness of tumor cells. In one such experiment, the CD44 splice isomer (CD44v6) was used to separate the CD44v6^+^ epithelial membrane antigen negative (EMA^-^) stem cell subtype from bladder tumors [33].

Further, the 67LR^+^CEACAM^-^ BCSCs were identified using two surface markers, namely, the 67kDa basal layer laminin receptor (67LR) and carcinoembryonic antigen related cell adhesion factor 6 (CEACAM6); 67LR is expressed in the junction of tumor stroma and found in 80% of high-grade invasive bladder cancer; CEACAM6 is a non-specific poor reaction antigen [33]. In both experiments, the stem cell subgroups with special tumor characteristics were isolated using cell surface markers that were normally expressed in the urinary epithelial basal cells.

ALDH1A1 is another widely used bladder cancer stem cells marker. By *in vitro* sphere formation assays and *in vivo* xenograft experiments, it was shown that ALDH1A1^+^ cells had better colony formation and tumorigenicity characteristics than ALDH1A1^-^ cells [34]. Moreover, the colony formation and tumorigenicity properties of BCSCs were significantly reduced by shRNA knockdown of the *ALDH1A1* gene. Also, the ALDH1A1^+^ cells were subtypes of CD44^+^ cells and may represent more primitive BCSCs [34].

Since the CSC markers are complex, BCSCs have also been isolated using other markers. For example, Li *et al.* combined 2 monoclonal antibodies of human bladder cancer against BCMab1 and CD44 to isolate the BCMab1^+^CD44^+^ subgroup with strong proliferative and self-renewal properties that were associated with stem cells [35].

#### Side population cell

The biological features of stem cells can also be used to isolate CSCs. Stem cells highly express the ATP-binding cassette transporter in order to pump out drugs. This characteristic has been utilized to sort CSCs based on their ability to efficiently efflux the DNA fluorescent dye Hoechst 33342 and isolate the side population cells by FACS. Compared to other cells, the side population cells demonstrate colony formation, self-renewal and multi-directional differentiation characteristics that are typical of CSCs [36].

## MOLECULAR MECHANISMS OF BLADDER CANCER STEM CELLS

Both normal stem cells and CSCs are characterized by their ability to self-renew. Signaling pathways related to the stemness maintenance and plasticity regulation of normal stem cells such as Hedgehog, Notch and Wnt are also involved in the stemness maintenance of CSCs including BCSCs. In addition, the tyrosine kinase receptor signaling pathway, interleukin-6 and tyrosine kinase 1 signaling pathways also play an important role in regulating the stemness of solid CSCs.

### Hedgehog signaling pathway

**Figure 2:**
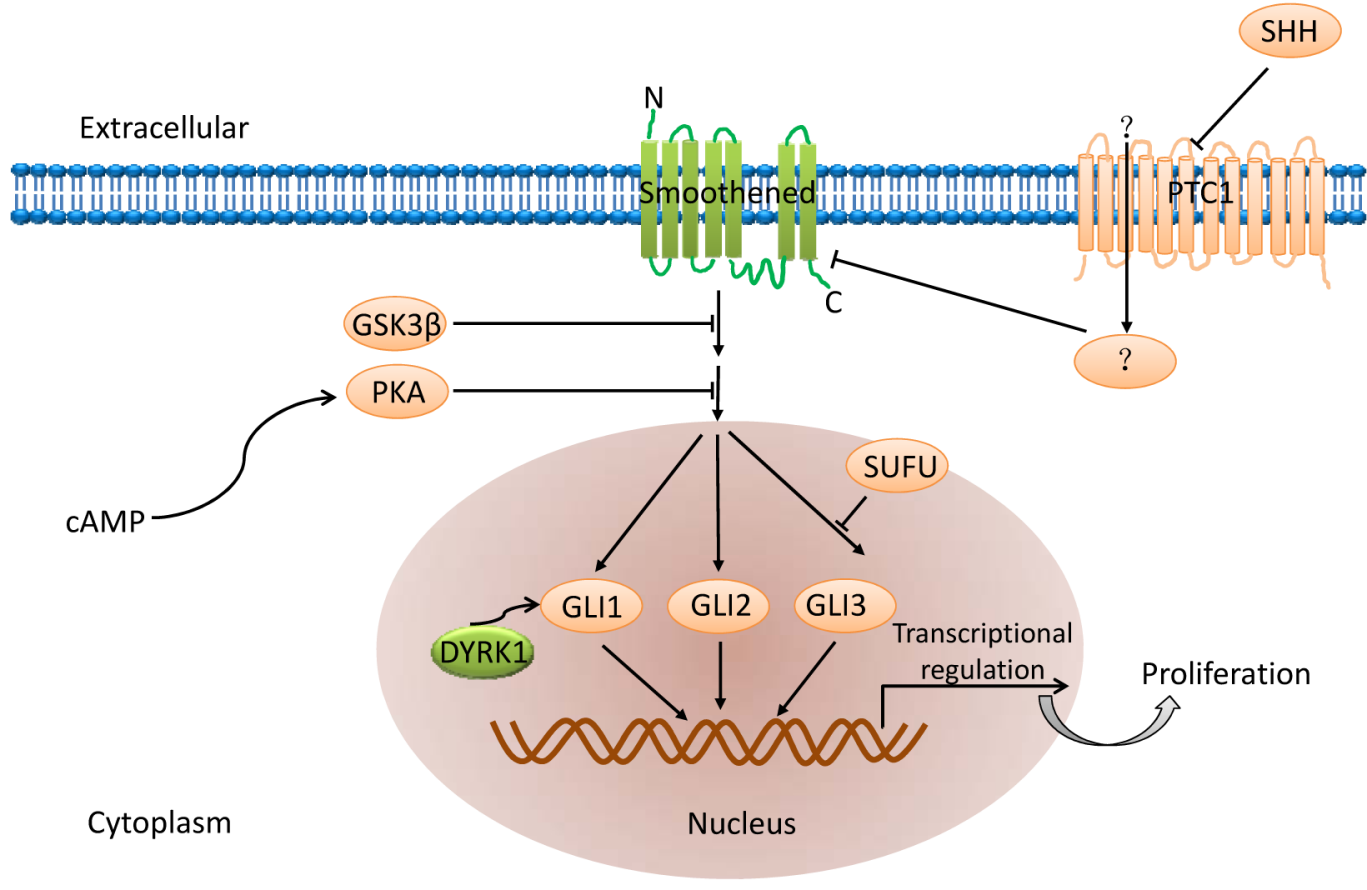
Hedgehog signaling pathway

Hedgehog signaling pathway (Figure 2) plays an important role in regulating embryogenesis and includes three homologous hedgehog genes namely Sonic Hedgehog (SHh), Indian Hedgehog (IHh) and Desert Hedgehog (DHh). Among these, SHh is highly expressed [37]. The hedgehog ligand binds to the transmembrane protein receptor Patched 1 (PTCH) and activates both the GLI transcription factor and the transmembrane protein receptor, Patched 1, which are inhibited by Smoothened (Smo) [38]. Aberrant activation of this pathway is central to development of a variety of tumors and their chemotherapy resistance [39]. Hence, inhibition of any of these factors may inhibit CSCs and therefore is a potential therapeutic target.

### Notch signaling pathway

**Figure 3:**
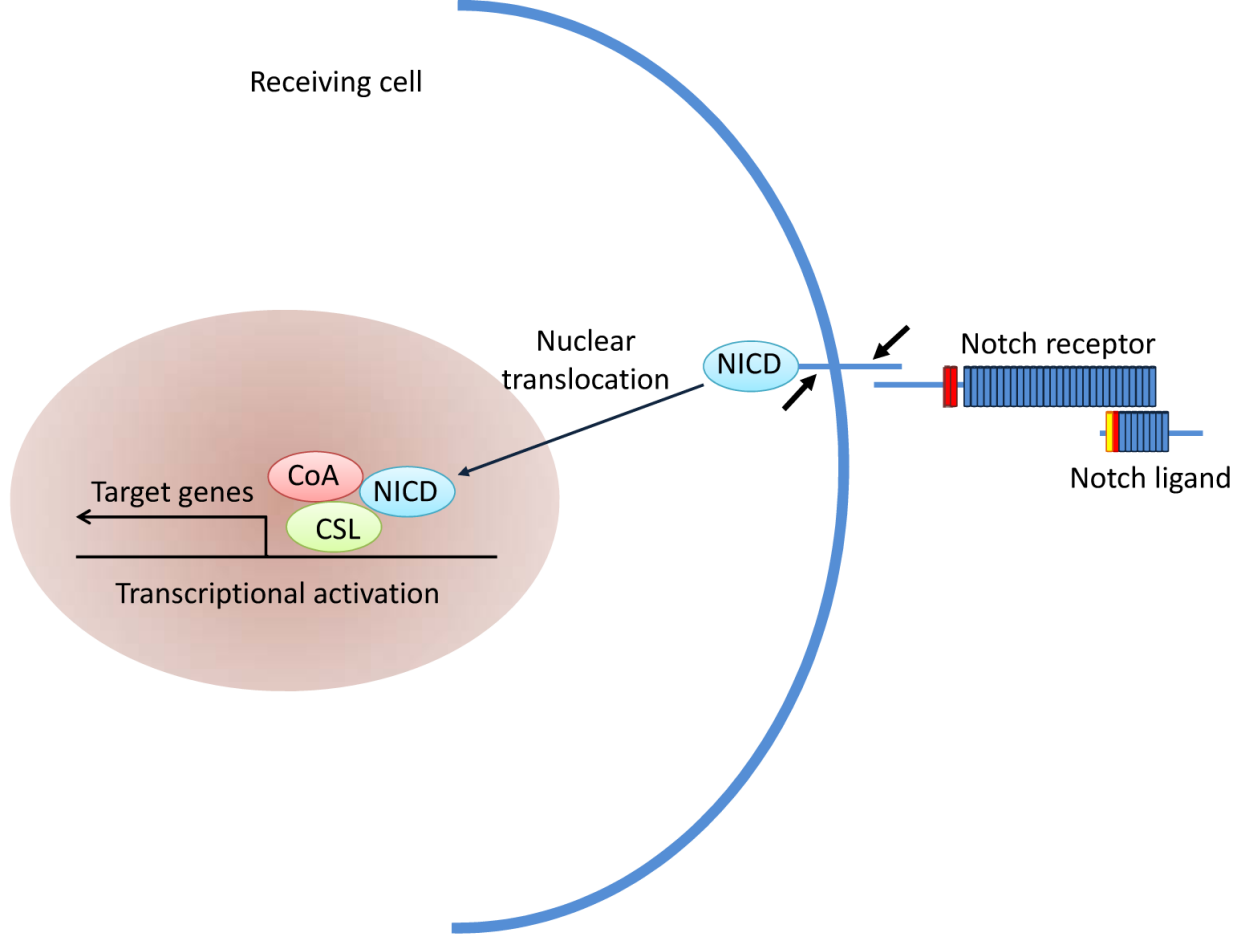
Notch signaling pathway

Notch signaling pathway (Figure 3) plays an important role in intercellular communication and cell fate determination during embryogenesis and in adult cells [40]. The pathway includes four receptors (Notch1∼Notch4) and five ligands that regulate expression of multiple target genes [41]. When the Notch ligand binds to its receptor, the extracellular and transmembrane domains of the receptor are degraded by metalloproteinases and the secretory enzyme, respectively, thereby releasing the notch intracellular domain [42]. The soluble intracellular domain is imported to the nucleus and activates its target genes. The Notch signaling pathway is hyperactivated in a variety of tumors and is involved in maintaining the stemness of CSCs [43]. In melanomas, Notch4 interacts with CSCs which express CD133 and ABCG2 to promote melanomagenesis [44]. In medulloblastoma, sustained high expression of Notch2 significantly increases the number of medulloblastoma stem cells [45-48], whereas the inhibition of Notch signaling decreases the medulloblastoma stem cells and suppresses carcinogenesis [49].

### Wnt signaling pathway

**Figure 4:**
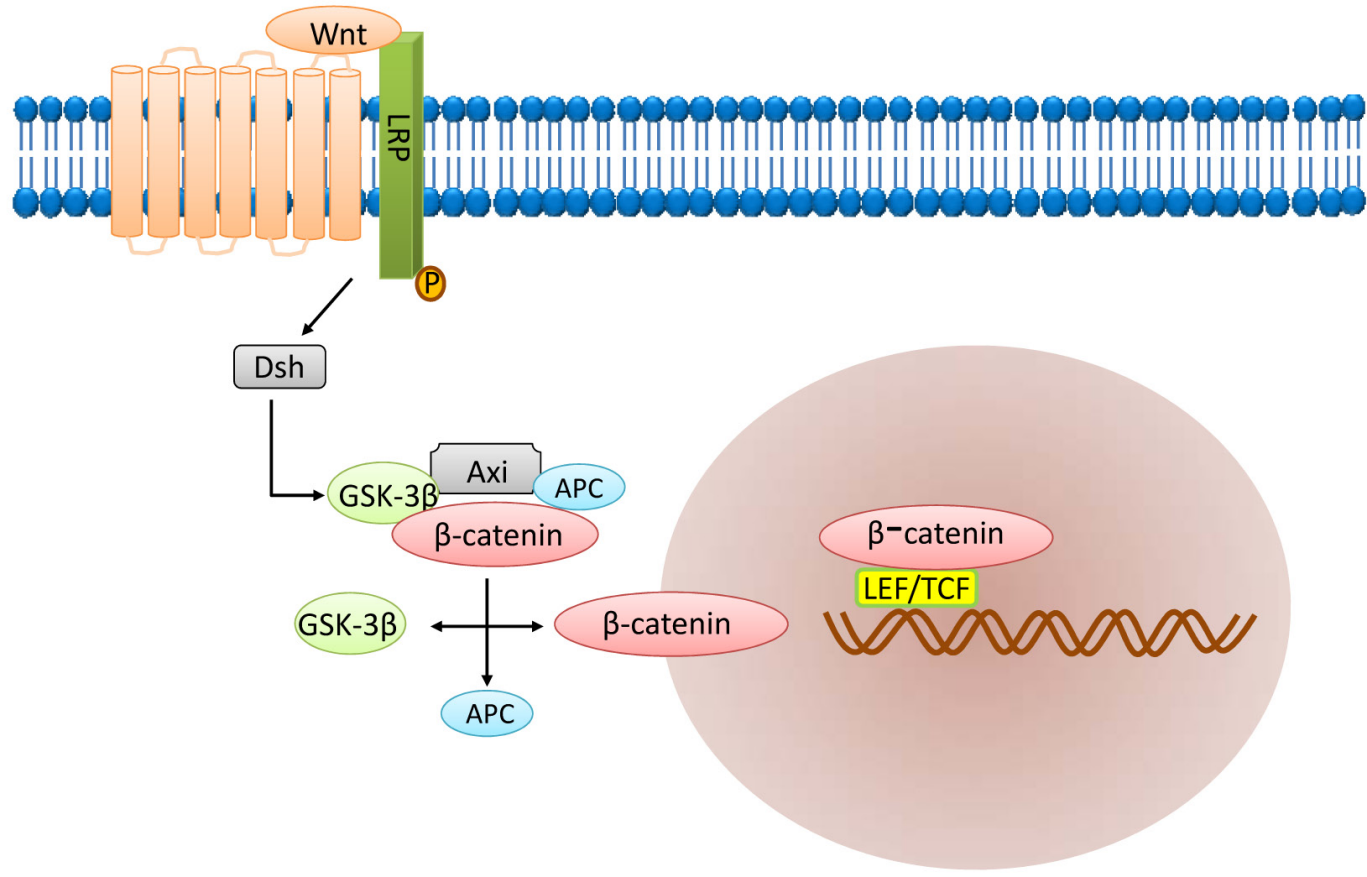
Wnt signaling pathway

The Wnt signaling pathway (Figure 4) is another important signaling pathway involved in embryogenesis and cell proliferation, survival and development [50, 51]. It is involved in stemness and maintenance of CSCs [52]. Wnt signaling maintains the stem cell pool and prevents their differentiation by stabilizing cytoplasmic β-catenin levels [53]. The main components of the Wnt signaling pathway include the extracellular factor Wnt, the transmembrane receptor, β-catenin, the “destruction complex”, and the transcription factor referred to as T cell factor (TCF). Wnt signaling is generally activated when both frizzled (FZD) and lipoprotein receptor-related protein (LRP) bind to Wnt.

After activation, the Wnt protein bound to the cell surface receptor FZD and LRP5/6 triggers intracellular signal transduction resulting in activation of the Dsh protein [54]. Activated Dsh then binds to Axin and Frat1 to form a complex with GSK-3β and APC. Then, Frat1 mediates the release of GSK-3β from Axin resulting in accumulation of dephosphorylated β-catenin. Dephosphorylated β-catenin is transported to the nucleus to activate the target genes including c-myc, stromelysin, fibroblast growth factor, epithelial cell growth factor and cyclin D1. Expression of these genes promotes tumorigenesis. Thus, tumorigenic β-catenin mutation results in aberrant Wnt signaling in the CSCs, thereby inducing tumorigenic proliferation [55].

### Other signaling pathways

Aberrant activation of the JAK-STAT signaling pathway has been shown to induce tumorigenesis [56, 57]. STAT3 activation is required for the tumorigenic functions of multiple CSCs [57-60]. IL-6 cytokine that is critical for the stemness of normal stem cells that also regulates CSCs by modulating *OCT2*, *CD44* and *SOX2* gene expression. The IL6/JAK/STAT3 signaling pathway helps maintain plasticity of breast CSCs and also activates mTORC1-STAT3 signaling pathway to maintain BCSC stemness [37, 41]. The tumorigenic tyrosine kinase receptor also contributes to stemness maintenance and chemotherapy resistance of non-small cell lung cancer stem cells [61].

## DRUG RESISTANT MECHANISM OF BCSCS

**Figure 5:**
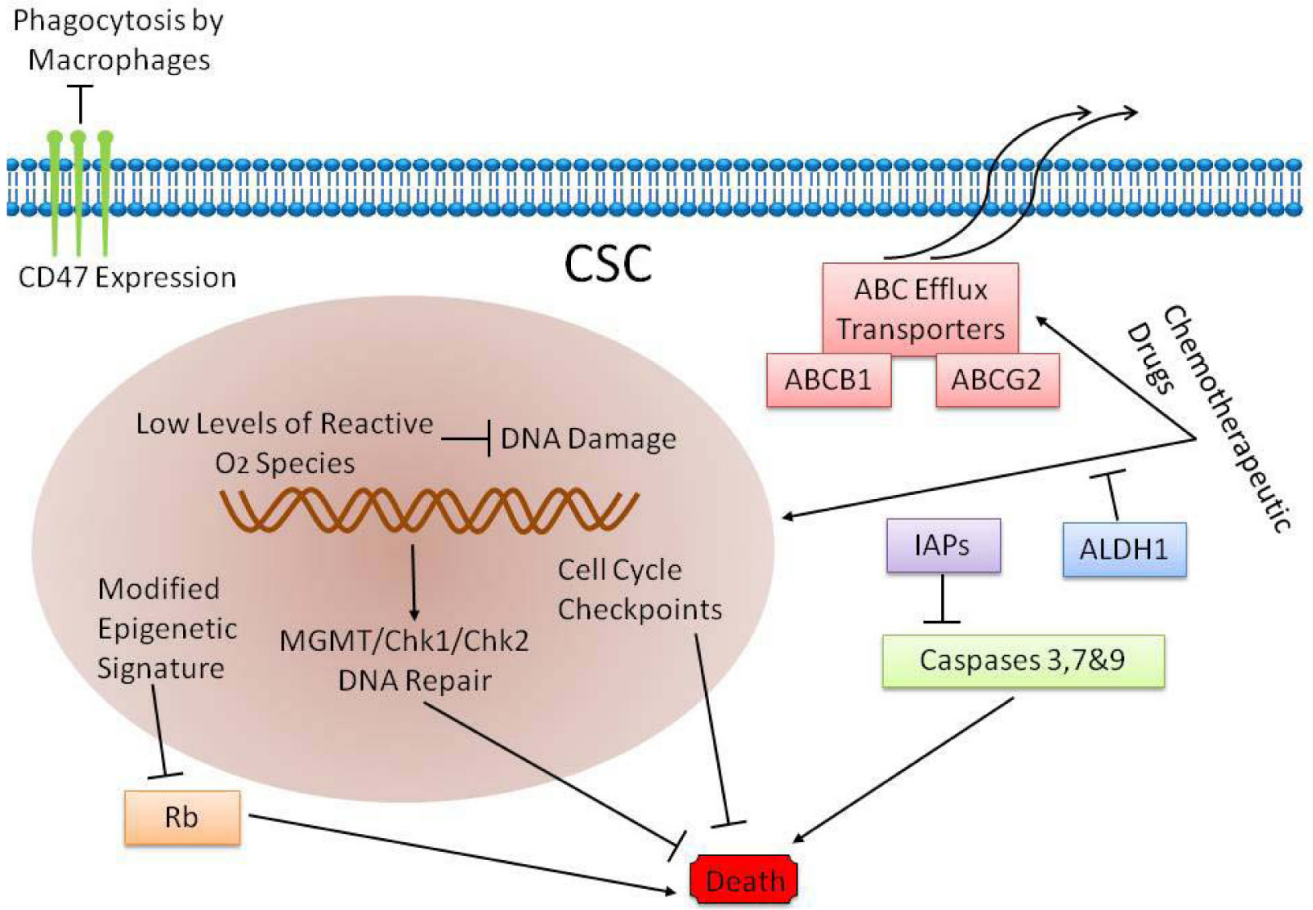
Drug resistant mechanism of BCSCs

The high recurrence rates of papillary bladder cancer as well as high invasiness and metastasis of myometrial invasive bladder cancer are attributed to BCSCs [12, 15, 62-64]. Also, recurrent bladder cancers are usually chemoresistant, although the primary cancer is sensitive to chemotherapy [65]. There are two possible reasons for the chemoresistance. First, the chemotherapeutic drugs are unable to access the core of the tumor [64]; second, the BCSCs are chemoresistant [65, 66]. In fact, chemotherapy in combination with stem cell targeted therapy is the most promising therapy currently available for bladder cancer [67]. Therefore, it is important to identify the mechanisms that contribute to chemoresistance of BCSCs and deciphere ways to suppress or eliminate it to improve the prognosis of bladder cancer patients. The mechanisms that contribute to chemoresistance mechanisms of bladder cancer stem cells are: (1) active pumping out of chemotherapy drugs by the BCSCs; (2) enzymatic breakdown of drugs and their metabolites rendering them ineffective; (3) inhibition of programmed cell death (apoptosis) and (4) regulation of cytokines and other immune substances that inhibit the anti-tumor immune response (Figure 5).

### ABC transport proteins

The ABC transport proteins in stem cells, especially the Vallazapine-sensitive ABCG2, pump out intracellular metabolites, drugs and toxic substances [68]. This was previously used as the principle for isolating the side population (SP) CSCs [69]. The SP cells sorted from T24 human bladder cancer cell lines showed significant colony-forming ability upon subculturing and were tolerant to radiotherapy and chemotherapy [36]. This suggested that the ABC transport proteins contributed to the chemoresistance of BCSCs.

### Acetaldehyde dehydrogenase (ALDH)

Acetaldehyde dehydrogenase (ALDH) is significantly overexpressed in BCSCs [70]. In xenograft studies of mouse colon cancer, the cyclophosphamide and its metabolites were oxidized and inactivated by ALDH, thereby helping tumor cells to survive chemotherapeutic treatment [71, 72]. Since ALDH is overexpressed in BCSCs, inactivation of chemotherapeutic agents and their metabolites by ALDH is a likely resistance mechanism for bladder cancer.

### Antioxidant enzymes

During radiotherapy and chemotherapy, reactive oxygen species (ROS) that are generated in tumor cells, irreversibly damage the DNA, thereby resulting in tumor cell death [73, 74]. In epithelial CSCs, ROS levels were significantly lower as a result of robust free radical scavenging system that protected the tumor cell DNA from endogenous or exogenous ROS and oxidative damage [75, 76]. Also, urinary epithelial CSCs upregulate antioxidant enzymes like superoxide dismutase (SOD2) and Hemeoxygenase, thereby decreasing oxidative DNA damage [77]. The presence of these antioxidant enzymes leads to a significant increase in the viability of CSCs during chemotherapeutic treatment.

### Apoptosis resistance

Urinary epithelial CSCs express high levels of IL11, IL18 and IL23 mRNA [70]. These interleukins promote *in vitro* survival and growth of tumor cells by activation of anti-apoptotic genes including cFLIP / FLAME-1 and Bcl-xL [78]. High interleukin levels have also been reported in prostate, breast, bladder and colorectal cancers [78]. IL23 is involved in the activation of the STAT-3 pathway, which promotes tumor survival, proliferation, invasion and angiogenesis [79].

Therefore, cytokines produced by the BCSCs enable them to survive chemotherapy and mediate recurrence. Therefore, to improve therapeutic outcomes, it is important to uncover the molecular mechanisms relevant for chemoresistance of BCSCs.

### Targeted therapy for bladder cancer CSCs

Drug resistance is a barrier to the effectual remedy of most cancers. Although CSCs-targeting treatment is still far away from clinical use, it is still widely believed to be a promising approach to drug resistance and may create better curative effects on cancers [80]. However, we need to identify CSCs-associated genes with a comprehensive study on the genomic profiles of CSCs in order to establish intervention targets. By single cell sequencing, Yang *et al* identified 21 key mutation genes in BCSCs including 6 genes,*ETS1*, *GPRC5A*, *MKL1*, *AWR*, *PITX2* and *RGS9BP* that were reported for the first time in bladder cancer. Besides, co-mutagenesis of ARID1A, GPRC5A and MLL2 by CRISPR/Cas9 technology significantly enhanced self-renewal and tumor initiation properties of BCSCs [19]. This study explored the genetic basis of human BCSCs and demonstrated their phylogeny. Besides, cancer stem cell function also interacts with epigenetics. Histone modification and chromatin rearrangement mutations usually exist in CSCs [81], promoting tumour initiation in different mechanisms [82]such as influencing the genome integrity [83]or leading to epigenetic reprogramming in iPS [84].

Analysis of the heterogeneity of different CD44^+^ subsets in bladder transitional cell carcinoma demonstrated many proteins that are involved in self-renewal such as nuclear Bmi-1, Stat3, and β-Catenin. Also, Activated Gli1 was found in 85% of CD44^+^ tumor cells suggesting that Gli1 was a potential molecular target [85].

The above mutations and abnormally expressed genes in CSCs are all potential therapeutic targets.

The monoclonal antibody therapy targeting tumor cell antigens is most effective among cancer treatments. Immunofluorescence and FACS analyses showed that CD47 is widely expressed in bladder urothelial carcinoma cells [32]. Compared to CD44^-^ tumor cell subsets, higher CD47 expression was observed in CD44^+^ tumor stem cells. CD47 is immunosuppressive and inhibits macrophage phagocytosis by interacting with SIRPα, a plasma membrane protein that is mainly expressed on bone marrow cells including macrophages, neutrophils, eosinophils and dendritic cells [86]. Treatment with monoclonal anti-CD47 antibody can induce phagocytosis of macrophages in bladder cancer cells *in vitro* and significantly reduce tumor growth in bladder cancer xenografts in a dose-dependent manner in the recipient mice [32, 86].

The CSC-targeted chemo-radiotherapy, which is different from conventional chemo-radiotherapy, combined with small molecule inhibitors that target CSC-specific signaling pathways are expected to be effective therapeutics in the future.

## CONCLUSIONS AND FUTURE PROSPECTS

The CSCs, which were first identified in human malignant blood tumors [87], have been subsequently identified in many solid tumors [10, 38, 88-90]. *In vivo* and *in vitro* experiments with tumor cells in genetically engineered mice has greatly advanced our understanding regarding cancer stem cell role, including insights regarding their relevance for clinical applications. However, clinical success from the point of view of CSCs needs further understanding and clinical development. Compared to CSCs in hematological malignant tumors, the solid tumor CSCs interact with a large number of endothelial cells, fibroblasts, and inflammatory cells. To some extent, CSCs isolated from solid tumors with one or several specific markers is not equal to the whole tumor mass [91]. Therefore, study and clinical applications regarding solid tumor CSCs need to consider cell-cell and cell-stroma interactions, which are highly complex and sometimes difficult to simulate. Also, the field of solid tumor CSCs is still developing and there is no consensus yet regarding the existence, origin and development mechanism of CSCs. These areas need robust scientific exploration.

In the 21st century, a variety of malignant tumors including bladder cancer have extremely poor prognosis. The CSCs that have been discovered in the past decade or two provide a novel perspective to anticancer therapy that needs to be pursued strongly. In regard to BCSCs, it is important to determine specific bladder cancer stem cells markers, isolate and identify the origin and role of different categories of BCSCs with self-renewal and differentiation ability. Greater understanding of the biology of BCSCs will provide important information regarding disease classification, prognosis, treatment and early intervention, which will all improve clinical outcomes. We believe that with the deep understanding of BCSCs, the diagnosis and treatment of bladder cancer will make great progress in the near future.
